# Renoprotective effects of apocynin and/or umbelliferone against acrylamide-induced acute kidney injury in rats: role of the NLRP3 inflammasome and Nrf-2/HO-1 signaling pathways

**DOI:** 10.1007/s00210-024-03271-9

**Published:** 2024-07-19

**Authors:** Saad A. Ageena, Adel G. Bakr, Hamada A. Mokhlis, Mohamed F. Abd-Ellah

**Affiliations:** 1 Department of Pharmacology & Toxicology, Faculty of Pharmacy, Al Azhar University, Assiut Branch, Assiut, 71524 Egypt; 2 Department of Pharmacology & Toxicology, Faculty of Pharmacy (Boys), Al-Azhar University, Cairo, Egypt; 3 Department of Pharmacy Practice, Faculty of Pharmacy, Kantara Branch, Sinai University, Cairo, Egypt

**Keywords:** Acrylamide, Umbelliferone, Apocyanin, Nrf2/HO-1, NLRP3, Nephrotoxicity

## Abstract

Acrylamide (ACR) is a toxic, probably carcinogenic compound commonly found in fried foods and used in the production of many industrial consumer products. ACR-induced acute kidney injury is mediated through several signals. In this research, we investigated, for the first time, the therapeutic effects of phytochemicals apocynin (APO) and/or umbelliferone (UMB) against ACR-induced nephrotoxicity in rats and emphasized the underlying molecular mechanism. To achieve this goal, five groups of rats were randomly assigned: the control group received vehicle (0.5% CMC; 1 ml/rat), ACR (40 mg/kg, i.p.), ACR + APO (100 mg/kg, P.O.), ACR + UMB (50 mg/kg, P.O.), and combination group for 10 days. In ACR-intoxicated rats, there was a significant reduction in weight gain while the levels of blood urea, uric acid, creatinine, and Kim-1 were elevated, indicating renal injury. Histopathological injury was also observed in the kidneys of ACR-intoxicated rats, confirming the biochemical data. Moreover, MDA, TNF-α, and IL-1β levels were raised; and GSH and SOD levels were decreased. In contrast, treatment with APO, UMB, and their combination significantly reduced the kidney function biomarkers, prevented tissue damage, and decreased inflammatory cytokines and MDA. Mechanistically, it suppressed the expression of NLRP-3, ASC, GSDMD, caspase-1, and IL-1β, while it upregulated Nrf-2 and HO-1 in the kidneys of ACR-intoxicated rats. In conclusion, APO, UMB, and their combination prevented ACR-induced nephrotoxicity in rats by attenuating oxidative injury and inflammation, suppressing NLRP-3 inflammasome signaling, enhancing antioxidants, and upregulating Nrf-2 and HO-1 in the kidneys of ACR-induced rats.

**Supplementary Information:**

The online version contains supplementary material available at 10.1007/s00210-024-03271-9.

## Introduction

Acute kidney injury (AKI) is a severe kidney disease condition characterized by a rapid increase in serum creatinine and/or a decrease in urine output. It occurs in approximately growing problems with undesirable health and economic effects (Koyner et al. 2014). Moreover, AKI is associated with complications of progressive chronic kidney disease, which leads to higher mortality rates (Chawla et al. 2014), and its incidence in the intensive care unit is about 52% (Ronco et al. 2019). It has been reported that various factors could contribute to the start and spread of AKI, including exposure to toxins like acrylamide (ACR) (Elhelaly et al. 2019).

Acrylamide is a chemical compound that is used in the production of many industrial and consumer products. It is also formed during cooking food at high temperatures above 120 °C, like potato chips and coffee. ACR has been linked to the development of AKI in some cases (May N. Bin-Jumah et al. 2021). There are no specific treatments for ACR-induced AKI. Interestingly, ACR has attracted the attention of researchers from different nations because of its possible risks to food. Several in vitro and in vivo studies have linked ACR to reproductive toxicity (Mokhlis et al. 2023), nephrotoxicity, hepatotoxicity (Kandemir et al. 2020), neurotoxicity, and possible carcinogenesis (Erkekoglu and Baydar 2014).

Interestingly, the mechanisms of ACR-induced AKI are not entirely understood. However, it has been suggested that ACR may cause nephrotoxicity by multiple signals such as oxidative stress injury and inflammatory perturbation in the kidney. ACR has been reported to induce oxidative stress injury by its active metabolite, glycidamide, and thus produce cytotoxicity (Nan et al. 2021). Besides, ACR increased reactive oxygen species (ROS) accumulation, changed the oxidant/antioxidant status, and caused organ damage (Cheng et al. 2015). Remarkably, the activation of ROS as a secondary messenger mediates NOD-like receptors (NLRs) and inflammasome activation (Nan et al. 2021). NLR family pyrin domain-containing3 (NLRP3) inflammasome consists of three proteins, namely, NOD-like receptor (NLR), adaptor protein ASC (apoptosis-associated speck-like protein containing a caspase recruitment domain (CARD)), and pro-caspase-1. Following the receptor’s detection of the stimulatory signal, ASC and pro-caspase-1 are recruited to form inflammasome, which activates caspase-1 and then cleaves pro-IL-1β and pro-IL-18 to produce biologically active inflammatory factors such as mature IL-1β and IL-18, respectively. Overall, this process is called pyroptosis (M. Zhao et al. 2023). Indeed, ACR has been reported to induce NLRP3 inflammasome activation, which triggers a series of inflammatory responses and specific tissue damage in rats (Alatshan and Benkő, 2021).

Interestingly, apocynin (APO) is a natural compound derived from the *Picrorhiza kurroa* and *Apocynum cannabinum* (Stefanska and Pawliczak 2008). It has anti-inflammatory, antioxidant, and immunomodulatory effects (Mahmoud et al. 2023; Hassanein et al. 2023; Bakr et al. 2022). Its antioxidant activity is due to its ability to inhibit the activation of the nicotinamide adenine dinucleotide phosphate NADPH oxidase enzyme complex, which plays a crucial role in the production of ROS (El-Sawalhi and Ahmed 2014). In addition, APO can down-regulate the expression of the NLRP3 inflammasome and alleviate renal fibrosis (Jin et al. 2019). Also, umbelliferone (UMB), also known as 7-hydroxycoumarin, is a natural compound found in various plants, such as Umbelliferae and Rutaceae families, with antioxidant, anti-inflammatory, and free radical scavenging (Hassanein et al. 2020, 2021b; Abdel-Wahab et al. 2022). Prior research has demonstrated the protective effects of UMB, such as anti-inflammatory activity against rheumatoid arthritis (Kumar et al. 2015) and hepatoprotective action against liver injury (Germoush et al. 2018). In addition, UMB has been reported to down-regulate the NLRP3 inflammasome activation and decrease inflammation of the kidney induced by gentamicin (Hassanein et al. 2021a).

There are no studies that reported the potential effects of APO and/or UMB on ACR-induced nephrotoxicity. Based on these findings, we hypothesize that APO and/or UMB can protect against the nephrotoxicity caused by ACR. We investigated the effects of APO and/or UMB on inflammation, oxidative stress, and pyroptosis in the kidneys caused by ACR-induced nephrotoxicity in rats.

## Materials and methods

### Reagents, chemicals, and kits

The reagents and chemicals supplier of acrylamide (ACR) and apocynin (APO) was Sigma-Aldrich, located in St. Louis, MO (USA). Umbelliferone (UMB) was obtained from Santa Cruz Biotechnology, Inc. (USA). Antibodies obtained from Biospes, China: anti-NLRP3 (Cat No. YPA1480), anti-ASC (Cat No. YPA1480), anti-GSDMD (Cat No. YPA2511), anti-caspase-1 (Cat No. YPA2348), anti-IL-1β (Cat No. YPA1070), anti-Nrf-2 (Cat No. YPA1865), anti-HO-1 (Cat No. YPA1919), and anti-β-actin (Cat. No. BTL1027) antibodies. Kits for real-time PCR; RNA extraction kit, complementary DNA synthesis kit, and SYBR Green obtained from (Vivants Technologies, Malaysia). The remaining chemicals were all analytical quality and came from certified local suppliers.

### Animals

Adult male Wistar albino rats, weighing between 190 and 210 g, were obtained from the animal care center of Assiut University in Egypt, where they were 12–14 weeks old. The animals were housed in controlled environments with a temperature of 23 ± 2 °C, a humidity of 60 ± 10%, and a light/dark cycle of 12/12 h. They were also allowed to roam freely and were fed a regular meal and tap water.

### Design of the experiment

Five groups of ten rats each were randomly selected from a pool of fifty rats in the following manner: Group I, the normal control group, was given a daily oral dose of 0.5% carboxymethyl cellulose (CMC) for 10 days. Group II: For 10 days, intraperitoneal administration of ACR (40 mg/kg/day) (Ghorbel et al. 2017) was used. Group III was administered ACR (40 mg/kg/day) intraperitoneally and APO (100 mg/kg/day; suspended in 0.5% CMC) (Cotter and Cameron 2003) orally for 10 days. For 10 days, Group IV was given ACR (40 mg/kg/day i.p.) and UMB (50 mg/kg; suspended in 0.5% CMC) orally once a day (Hassanein et al. 2021a). Group V: for 10 days, ACR (40 mg/kg/day i.p.), APO (100 mg/kg/day), and UMB (50 mg/kg/day) were administered orally with an hour between dosages to prevent drug interactions.

### Preparation of tissue and serum

Blood samples were taken using the cardiac puncture technique on the 11th day of the experiment, 12 h after the last dose, and while under anesthesia with 87 mg of ketamine/kg and 13 mg of xylazine/kg (Van Pelt 1977). Rat serum was separated by centrifugation at 5000 rpm for 10 min at 4 °C and used for estimation of serum electrolytes (sodium, potassium, and magnesium) as well as serum levels of urea, creatinine, and uric acid. The kidneys were removed and split into three sections after being cleaned with regular saline. For the qRT-PCR estimate, the first was kept at − 80 °C in RNA Later® (Ambion, USA). For western blot analysis, the second part was kept at − 80 °C in RIPA buffer (Biospes, China) with a protease inhibitor cocktail (Biospes, China). For histological analysis, the third piece was preserved in 10% formalin-buffered saline.

### Kidney function biomarkers

Serum electrolytes (sodium, potassium, and magnesium) as well as serum levels of urea, uric acid, and creatinine were assessed according to manufacturer instructions.

### Histopathological examination

The kidney tissue was embedded in paraffin blocks, dried, and fixed in saline buffered with 10% formalin. Hematoxylin and eosin (H&E) were applied to Sects. (4–5 µm) using the previously reported protocol by Suvarna et al. (2018). The sections were subjected to a blind examination using an Olympus light microscope (USA). Each group’s kidney tissues’ histopathological alterations were graded using the Derelanko (2017) methodology. The rating system was applied as follows: normal appearance ( −), mild ( +), moderate (+ +), and severe (+ + +).

### Renal oxidative stress biomarker estimation

An estimation of the content of renal reduced glutathione (GSH) was made using Ellman’s previously published methodology (Ellman 1959). The method of Marklund (1984) was used to determine the activity of renal superoxide dismutase (SOD). The technique of Uchiyama and Mihara (1978) was used to measure the renal malondialdehyde (MDA) level to determine lipid peroxidation. 

### Determining the TNF-α and KIM-1 levels by ELISA

Kidney tissue levels of kidney injury molecule-1 (KIM-1) and TNF-α were measured by using ELISA kits from ELK Biotechnology (Wuhan, China) (96 tests) according to the manufacturer’s instructions and based on the previously described principle (Van Weemen and Schuurs 1971).

### qRT-PCR analysis

To carry out real-time quantitative PCR (qRT-PCR), the total RNA extraction process was conducted using ice-cold reagents. A high-quality total RNA sample from kidney tissue was obtained by employing an RNA extraction as per the manufacturer’s guidelines. The RNA quality was assessed by measuring the 260/280 ratio. As directed by the manufacturer, a complementary DNA synthesis kit was used to synthesize the first strand of complementary DNA from 1500 ng of total RNA. Applied Biosystem Step One Plus (USA) real-time thermal cycler was used to amplify complementary DNA. Prepare the master mix, which consists of 2 µl of complementary DNA, 0.5 µl of each primer (1 µM), 7.5 µl of SYBR Green, and 4.5 µl of nuclease-free water. Make up the 15 µl mixture used for the amplification reaction. The amplification of complementary DNA was done using a real-time thermal cycler (Applied Biosystem Step One Plus, USA). The amplification reaction (15 µl) mixture contains 2 µl of cDNA, 0.5 µl of each primer (1 µM), 7.5 µl SYBR Green universal master mix, and 4.5 µl of nuclease-free water. As per one cycle, Table 1 lists the primers used. The 2^−△△Ct^ was used to analyze the outcome data and determine relative expression in accordance with Livak and Schmittgen’s (2001) methodology. GAPDH was employed as the endogenous reference gene.

**Table 1:** Primer sequences

Targeted gene	Direction and Sequence
Nrf2	F: 5′- ATTGCTGTCCATCTCTGTCAG-3′ R: 5′- GCTATTTTCCATTCCCGAGTTAC-3′
HO-1	F: 5′—TGCTTGTTTCGCTCTATCTCC-3′ R: 5′—CTTTCAGAAGGGTCAGGTGTC-3′
GAPDH	F:5′-TGCTGGTGCTGAGTATGTCG-3′ R:5′-TTGAGAGCAATGCCAGCC-3′

### Western blotting

The renal tissues were subjected to homogenization in cold RIPA buffer at 4 °C for 30 min. After homogenization, centrifugation was performed at 10,000 × g for 10 min at 4 °C to eliminate any tissue residue. The total protein concentration for each sample was determined according to the methods described by Bradford (1976). Subsequently, an equal amount of total protein (30 mg) was loaded onto a 10% SDS-PAGE gel for electrophoresis. The separated proteins were then transferred from the gel to a PVDF membrane (Millipore, Biospes, China) using the wet transfer method (Towbin et al. 1979). The membrane was then blocked for one hour at room temperature using 3% skim milk in tris-buffered saline-tween-20 buffer. Next, anti-NLRP3, anti-ASC, anti-GSDMD, anti-caspase-1, anti-IL-1β, anti-Nrf-2, and anti-HO-1 antibodies 1:1000 were added to the membrane and incubated for an additional night at 4 °C. Following that, the membranes were treated with a suitable secondary antibody, dilution 1:3000 conjugated to alkaline phosphatase for 1 h. Using a particular BCIP/NBT detection kit, the generated protein bands were visible (Genemed Biotechnologies, USA). For normalization, β-actin was utilized as the internal control. The protein bands obtained were subjected to densitometry analysis using ImageJ® software (National Institutes of Health, Bethesda, USA) to quantify the intensity of the bands.

### Statistical analysis

Each result is given as the means ± standard error of the means (S.E.M.). A one-way analysis of variance (ANOVA) was used to assess the overall significance of the differences in data. The software program GraphPad Prism (Version 8) was used to analyze the data. *P*-value < 0.05 was deemed appropriate for statistical significance, and multiple comparisons were adjusted using the proper Tukey–Kramer correction.

## Results

### The impact of APO and/or UMB on the kidney weight, final body weight, and kidney/body weight ratio

Data in Table 2 shows that injection of ACR at a dose of 40 mg/kg i.p. significantly reduced the final body weight of animals and caused no significant change in the kidney weight or kidney/body weight ratio compared to the control group. On the other hand, oral administration of APO, UMB, and combination kept the weight of animals, with slight increases in the final body weight in comparison with the ACR group.

**Table 2:** Effect of ACR and the treatment with APO, UMB, and their combinations on body weights and kidney weights of the rats in the experimental groups at the beginning and end of the experiment

Parameters	Normal	ACR	ACR + APO	ACR + UMB	COMB
Initial body weights (g)	135.4 ± 7	145.6 ± 9.8	146.3 ± 6.5	164.1 ± 6.5	151 ± 5.7
Final body weights (g)	168.4 ± 5.4	142.5 ± 8.6^a^	161.6 ± 6.4	166.3 ± 4.5	161.8 ± 5.8
Weight change (g)	33.0 ± 2.15	-2.5 ± 7.19^a^	15.29 ± 5.59	2.14 ± 2.37^a^	10.83 ± 4.72^a^
kidney weights (g)	1.26 ± .07	1.027 ± .076	1.22 ± .07	1.22 ± .04	1.15 ± .065

Data are expressed as mean ± S.E.M

^a^Significantly different from normal control group at *P* < 0.05

^b^Significantly different from ACR group at *P* < 0.05

^c^Significantly different from ACR + APO + UMB group at *P* < 0.05

### Impacts of APO, UMB, and their combinations on renal function biomarkers

Investigated renal function by measuring serum urea, creatinine, and uric acid, compared to the control group, was significantly greater in the ACR group. In contrast, treatment with APO, UMB, and their combination significantly decreased the level of serum urea, creatinine, and uric acid compared to the ACR group. Notably, the combination of APO and UMB exhibited a higher effect on normalizing renal function (Table 3). Additionally, results show that the protein expression of KIM-1 was markedly elevated in the ACR group compared to the normal control group. However, treatment with APO, UMB, and their combinations significantly reduced the renal content of KIM-1 compared to the ACR group. We observed that APO and UMB combinations restored renal KIM-1 content nearly to the normal level.

**Table 3:** Effect of apocynin, umbelliferone, and their combinations on renal function parameters and serum electrolyte in acrylamide-induced nephrotoxicity

Parameters	Normal	ACR	ACR + APO	ACR + UMB	COMB
Urea (mg/dl)	21.72 ± 0.87	58.56 ± 1.73^a^	36.88 ± 0.8^abc^	35.57 ± 0.70^abc^	30.63 ± 0.73^ab^
Uric acid (mg/dl)	1.5 ± 0.105	3.13 ± 0.084^a^	2.05 ± 0.09^abc^	1.83 ± 0.135^b^	1.58 ± 0.128^b^
Creatinine (mg/dl)	0.602 ± 0.03	1.45 ± 0.04^a^	0.89 ± 0.03^abc^	0.87 ± 0.03^abc^	0.67 ± 0.02^b^
KIM-1 (pg/mg protein)	84.31 ± 4.1	454.4 ± 11^a^	158.7 ± 4.9^abc^	202 ± 4^abc^	123.2 ± 5.4^ab^
Serum sodium (mEq/L)	156 ± 0.4	148.8 ± 2.6	160.7 ± 2.4	158.8 ± 2.2	163.1 ± 1.3
Serum potassium (mEq/L)	4.2 ± 0.16	7.5 ± 0.10^a^	6.3 ± 0.11^abc^	5.5 ± 0.08^ab^	5.3 ± 0.08^ab^
Serum magnesium (mg/dL)	2.9 ± 0.02	5.6 ± 0.08^a^	4.7 ± 0.06^abc^	4.2 ± 0.06^abc^	3.6 ± 0.08^ab^

Data are expressed as mean ± S.E.M

^a^Significantly different from normal control group at *P* < 0.05

^b^Significantly different from ACR group at *P* < 0.05

^c^Significantly different from ACR + APO + UMB group at *P* < 0.05

### Impact of APO, UMB, and their combinations on serum electrolytes of ACR-intoxicated rats

There was no significant variation between groups in serum Na concentration (hypo or hypernatremia); on the other hand, hyperkalemia and hypermagnesemia were observed in ACR group rats. Interestingly, administering APO, UMB, or both greatly reduced the elevation of serum K and Mg. Notably, the combination of APO with UMB exhibited a significant reduction in serum K and Mg as compared to each drug alone (Table 3).

### Impact of APO, UMB, and their combinations on histopathological changes in ACR-induced AKI

**Fig. 1:**
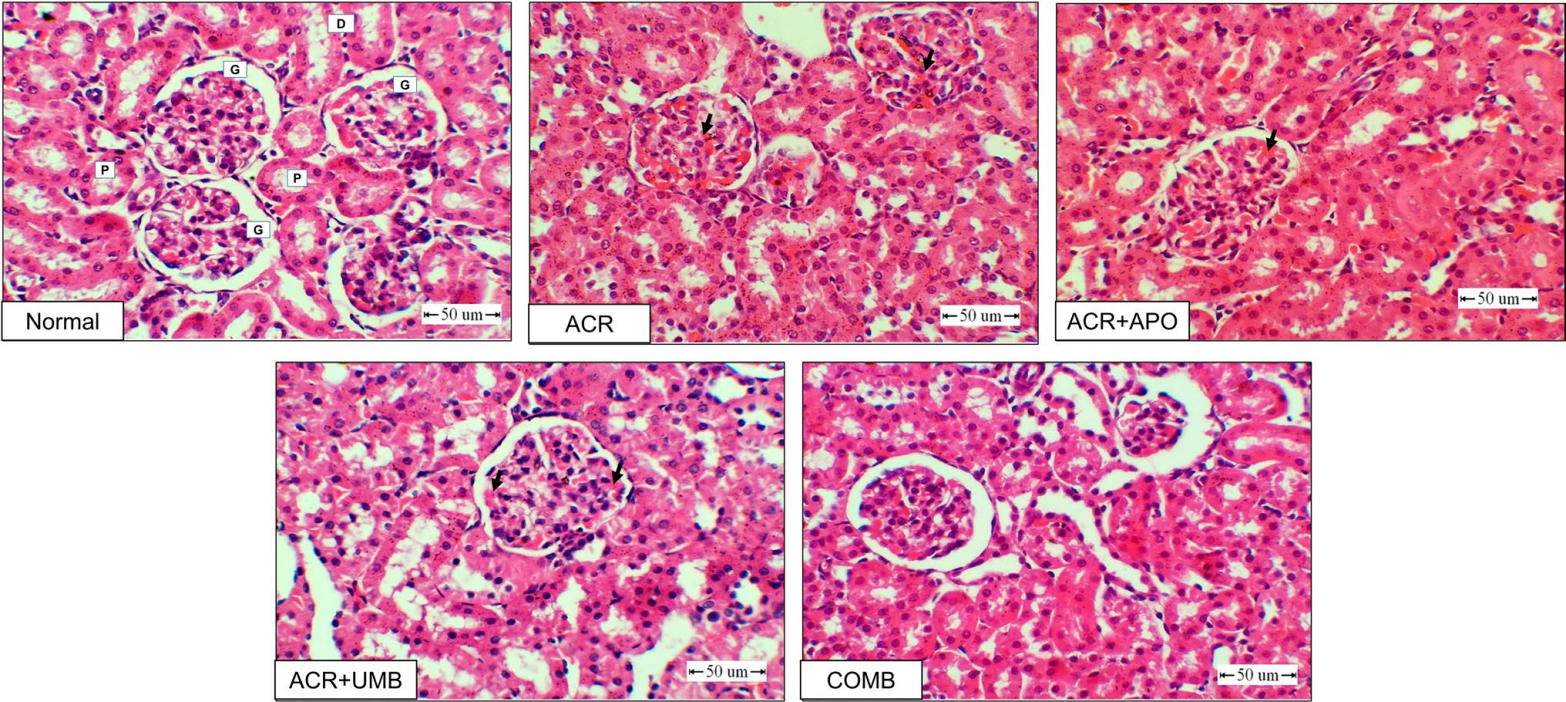
Photomicrographs of renal sections stained with H&E (400 ×). (Normal) A kidney section from the normal control group shows healthy glomeruli (G), proximal (P), and distal convoluted tubules (DI). (ACR) A kidney section from the ACR group shows damage to proximal and distal convoluted tubules, hyperemia of the capillary tufts and blood vessels, and interstitial hemorrhage (thick arrow). (APO) A kidney section from the group treated with APO shows moderate damage to proximal and distal convoluted tubules, moderate hyperemia of the capillary tufts and blood vessels, and moderate interstitial hemorrhage. (UMB) A kidney section from the group treated with UMB shows mild damage to proximal and distal convoluted tubules, hyperemia of the capillary tufts and blood vessels, and interstitial hemorrhage. (COMB) A kidney section from rats treated with APO + UMB shows a normal histological structure

Figure 1 shows the investigated histopathological sections stained with H&E for kidney tissues. In the normal control group, renal tissue had a normal histological structure with no histopathological changes. In the ACR group, there were extensive histopathological changes in the form of hyperemia of the capillary tufts. Marked dilation and hyperemia of the blood vessels and thickening of their tunica media were observed. A marked interstitial hemorrhage expressed by extravasated red blood cells was noticed. The APO group had marked hyperemia of the capillary tufts and showed marked dilation, hyperemia of the blood vessels, and thickening of the tunic media. A marked interstitial hemorrhage was also noticed. The UMB-treated group showed slight hyperemia of the capillary tufts. A mild interstitial hemorrhage was observed. The combination of APO and UMB had a remarkably healthy appearance of the tissue and a decrease in the intensity of the blood vessel changes observed in other treated groups (Fig. 1).

There are marked vascular changes, hyperemia of the capillary tufts and blood vessels, and interstitial hemorrhage in tissues collected from the ACR group. At the same time, APO provided mild protection against these changes, while UMB had moderate protection. Collectively, the combination of both APO and UMB had remarkable improvements in the alterations due to ACR-induced AKI, as shown in Table 4.

**Table 4:** Histopathological lesions scores (H&E)

Histopathological lesions	Normal	ACR	ACR + APO	ACR + UMB	COMB
Damage of proximal and distal convoluted tubules	−	+ + +	+ +	+	−
Hyperemia of the capillary tufts and blood vessels	−	+ + +	+ +	+	−
Interstitial hemorrhage	−	+ + +	+ +	+	−

**( −)** no change

(** +)** mild change

**(+ +)** moderate change

**(+ + +)** severe change

### Impact of APO, UMB, and their combination on renal oxidative stress biomarkers

**Fig. 2:**
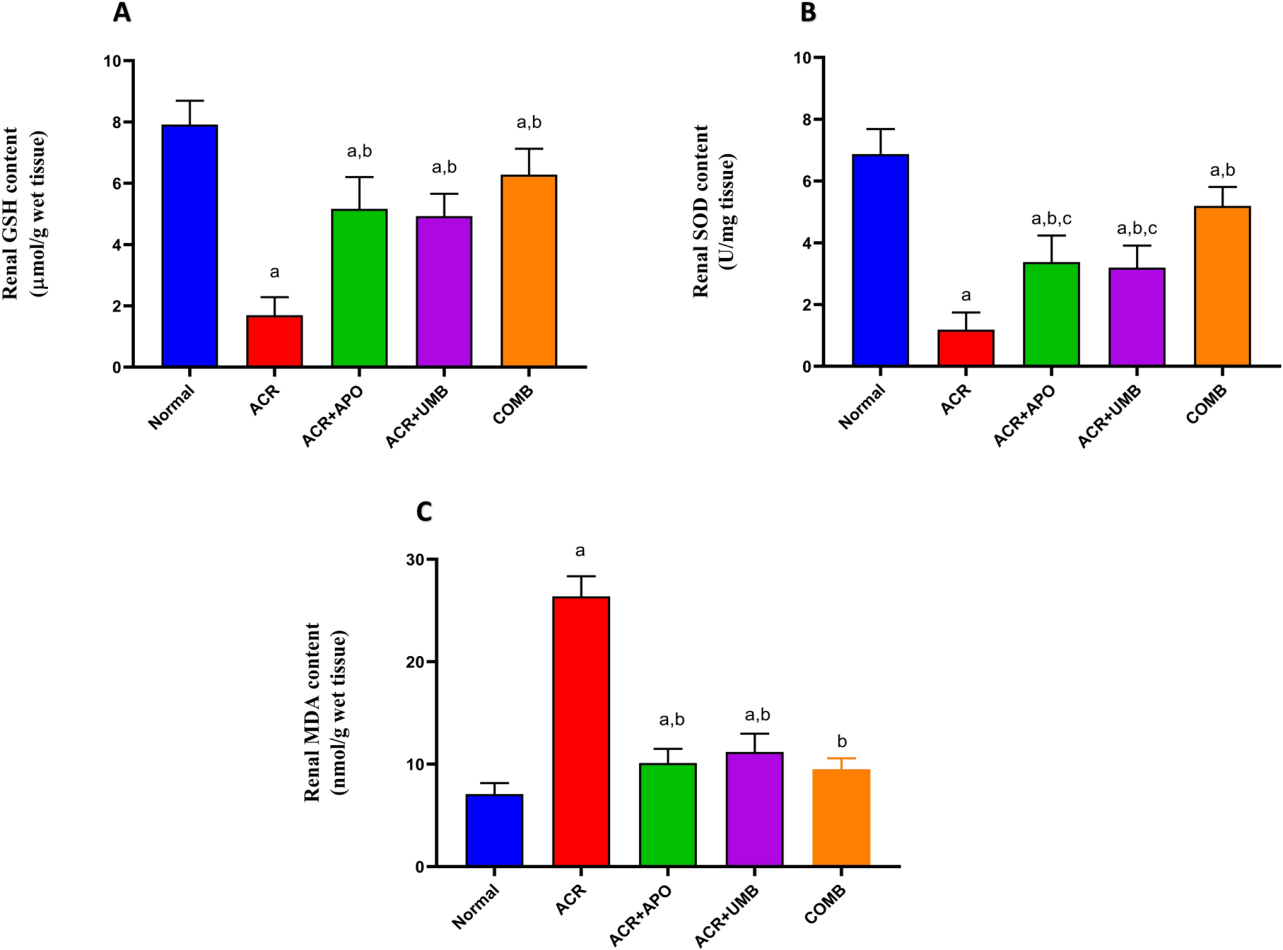
Effect of APO, UMB, and their combinations on oxidative stress and antioxidant biomarkers (MDA, GSH, and SOD) in renal tissue homogenate activity against ACR-induced AKI. ^a^Significantly different from the normal control group at *P* < 0.05, ^b^Significantly different from the ACR group at *P* < 0.05, ^c^Significantly different from the COMB group at *P* < 0.05

Figure 2 demonstrates that ACR administration caused a significant reduction in renal GSH and SOD levels when compared to normal control rats. Conversely, a significant increase in renal MDA levels was noted in ACR rats relative to their normal control. In comparison to the ACR group, treatment with APO, UMB, and their combination significantly elevated the GSH and SOD levels while MDA concurrently decreased levels.

### Impact of APO, UMB, and their combinations on mRNA and protein expression of Nrf-2 and HO-1

**Fig. 3:**
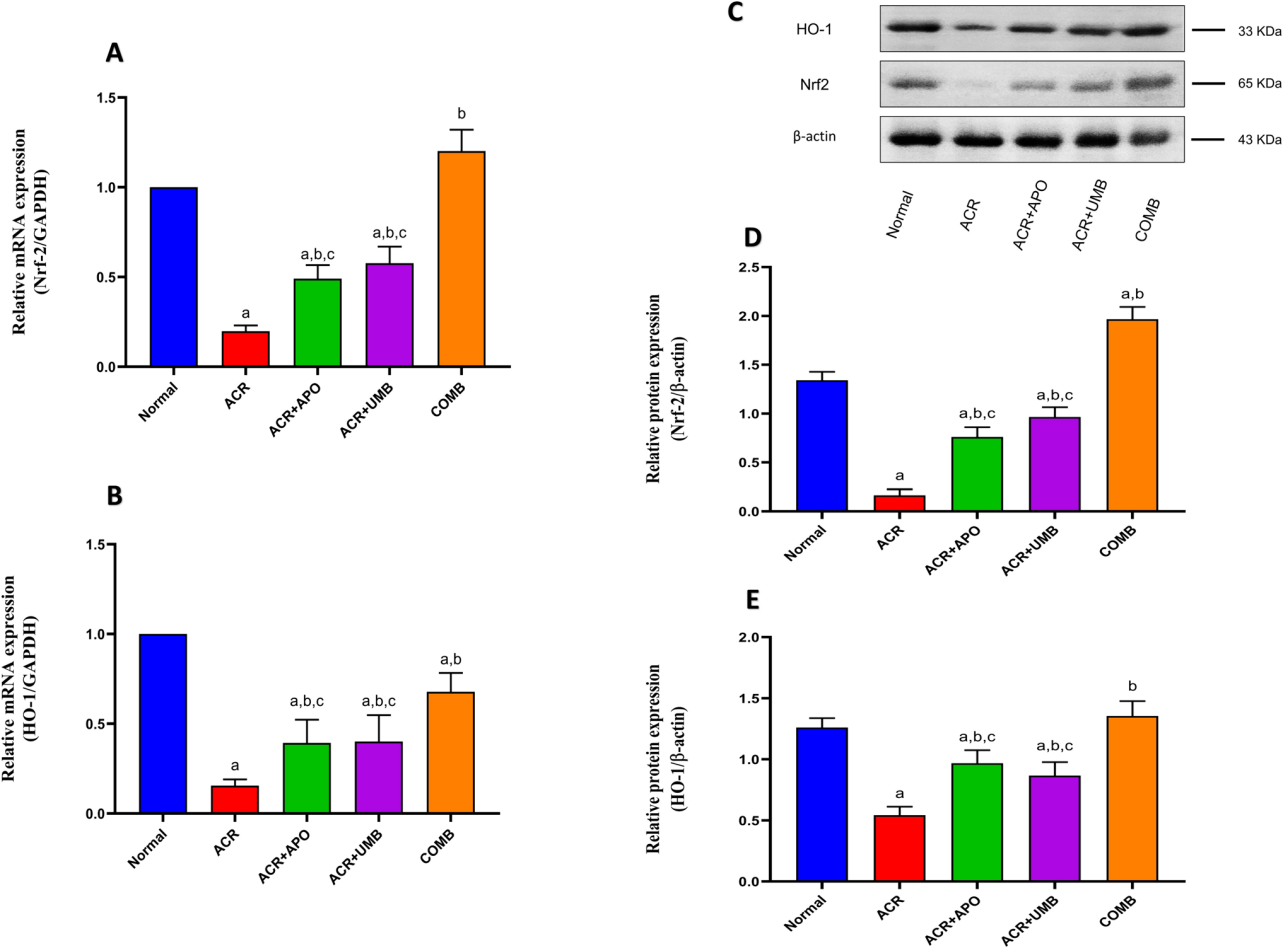
Effect of APO, UMB, and their combinations on the renal mRNA expression and protein expression of Nrf-2 and HO-1 against ACR-induced AKI. Quantitative RT-PCR was used for the determination of mRNA expression, and western blot analysis was used for the determination of protein expression. ^a^Significantly different from the normal control group at *P* < 0.05. ^b^Significantly different from the ACR control group at *P* < 0.05. ^c^Significantly different from the COMB group at *P* < 0.05

Significant changes were seen in the renal protein and mRNA expression of Nrf2 and HO-1. The renal protein and mRNA expression of Nrf2 and HO-1 showed a significant down-regulation in the ACR group compared to the normal group. However, treatment with APO and UMB significantly restored both protein and gene expression of renal Nrf2 and HO-1 compared to the ACR group. In parallel, the combination group shows higher efficacy in normalizing Nrf-2 and HO-1 protein and mRNA expression levels (Fig. 3).

### Impact of APO, UMB, and their combinations to suppress the pro-inflammatory cytokines TNF-α

**Fig. 4:**
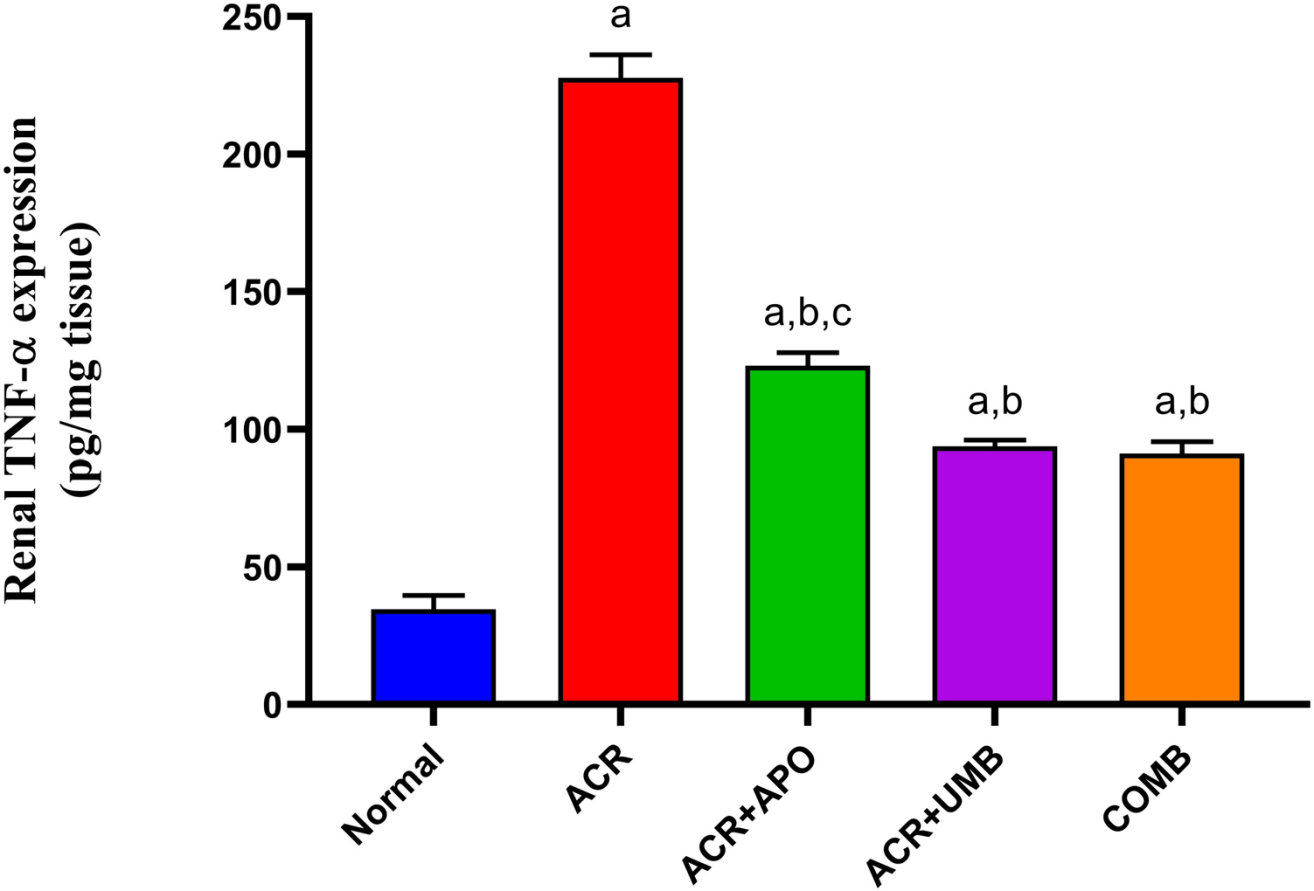
Effect of APO, UMB, and their combinations on TNF-α expression against ACR-induced AKI. ELISA kits were used for the determination of protein expression. ^a^Significantly different from the normal control group at *P* < 0.05. ^b^Significantly different from the ACR group at *P* < 0.05. ^c^Significantly different from the COMB group at *P* < 0.05

Furthermore, we explored how APO and UMB influenced proinflammatory TNF-α in ACR-induced AKI. When compared to control rats, ACR caused a significant rise in TNF-α protein expression. Treatment with APO, UMB, and their combinations significantly reduced TNF-α protein expression compared to the ACR group. It is worth noting that, as compared to each medication alone, co-treatment with APO and UMB dramatically reduced TNF-α expression (Fig. 4).

### Impact of APO, UMB, and their combinations on the NLRP-3 inflammasome signaling pathway

**Fig. 5:**
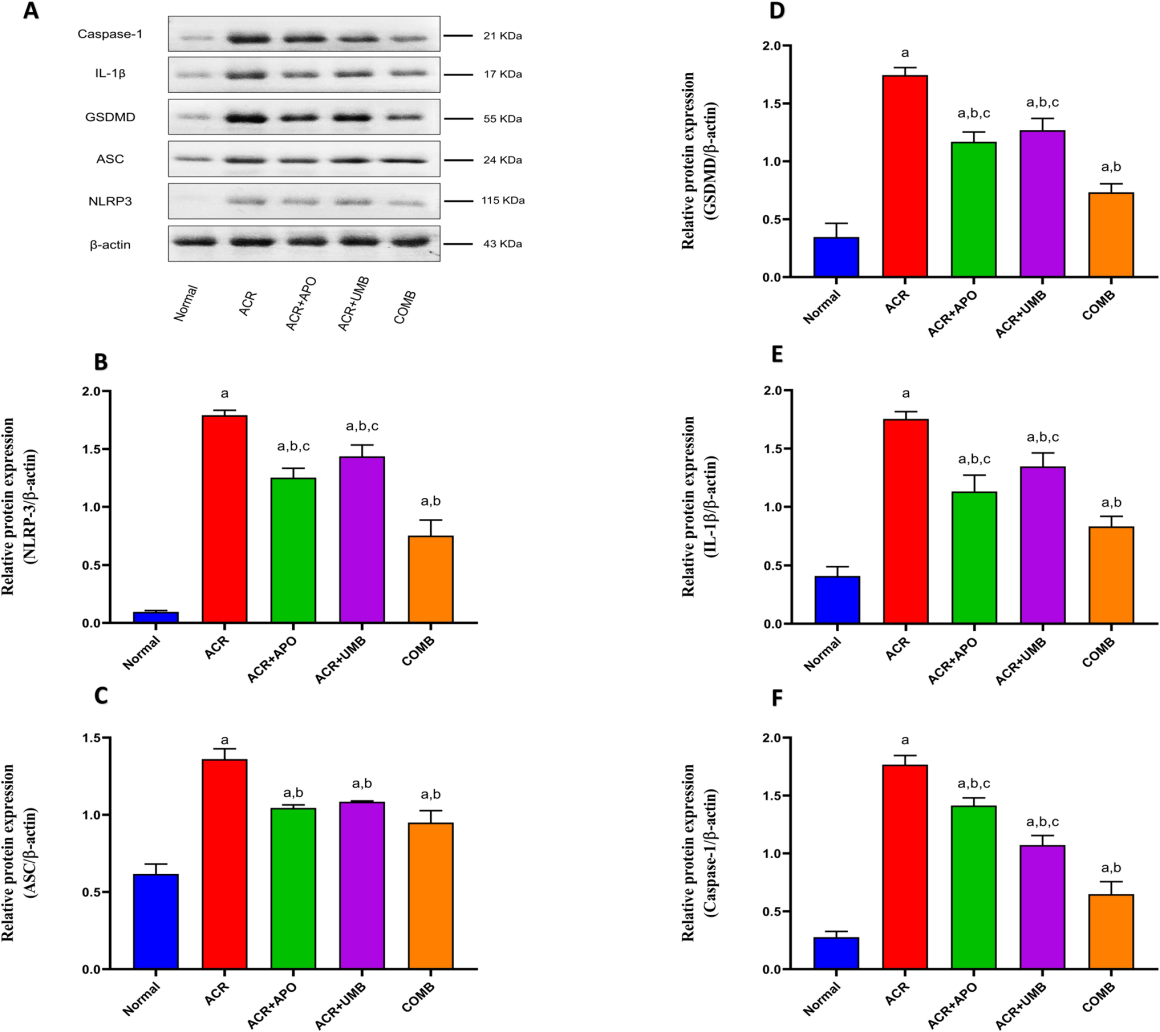
Effect of APO, UMB, and their combinations on renal expressions of NLRP-3, IL-1β, GSDMD, and ASC against ACR-induced AKI. Western blot analysis was used for the determination of the protein expression of these markers. ^a^Significantly different from the normal control group at *P* < 0.05. ^b^Significantly different from the ACR group at *P* < 0.05. ^c^Significantly different from the COMB group at *P* < 0.05

Statistical analysis revealed a significant increase in the protein expression of NLRP3, ASC, GSDMD, IL-1β, and caspase-1in ACR-treated rats as compared with control rats. Treatment with APO, UMB, and their combination showed significantly decreased expression of NLRP3, ASC, GSDMD, IL-1β, and caspase-1 as compared to ACR-treated rats. Furthermore, we observed that the suppression of the NLRP-3 signaling pathway is more pronounced when APO and UMB are combined (Fig. 5).

## Discussion

Acrylamide is a chemical compound utilized in numerous industries and created during cocking at high temperatures. Occupational and cumulative toxicities are observed (Guo et al. 2020; Exon 2006). ACR toxicity causes multiple toxicities, such as neurotoxicity, cardiotoxicity, reproductive injury, and nephrotoxicity (Wang et al. 2022a; Sengul et al. 2021; Wang et al. 2022b). Apoptosis, oxidative stress, and inflammation are the key mechanisms by which ACR is harmful (Song et al. 2021).

Natural chemicals such as APO and UMB were used in this study. They are therapeutically promising for evaluating the possible protective effects against ACR-induced AKI. Previous studies demonstrated that APO and UMB have antioxidant and anti-inflammatory qualities, as well as the ability to scavenge free radicals (Hassanein et al. 2023; Sami et al. 2022; Abd El-Ghafar et al. 2021). In our study, the toxicity of ACR administration resulted in a significant decline in body weight gain. This reduction in body weight gain may be the result of ACR’s potent appetite-suppressing effects, which lead to a decrease in food consumption (Guo et al. 2020).

The renal function biomarkers evaluated in the current research—serum urea, creatinine, and uric acid levels—were notably greater in the ACR-treated group, in addition to disturbance in the electrolyte balance (Na^+^, K^+^, and Mg^2+^); however, treatment with APO, UMB, and their combination resulted in a notable decline in serum urea, creatinine, and uric acid, restored electrolyte balance. Prior studies have linked the generation of ROS to tubular and glomerular damage, which raises kidney functioning (Lujia Zhang 2023; Wang et al. 2022a; Sengul et al. 2021). Also, ACR-induced AKI involves structural damage in the kidney, specifically tubular injury (Ibaokurgil et al. 2023). KIM-1 is a transmembrane protein that is an early biomarker of AKI due to its upregulation in renal tubular injury and inflammation. Downregulating KIM-1 expression may indicate a reduction in renal damage (Tanase et al. 2019). On the other hand, renal tubular and glomerular damage caused by ACR and its metabolite was effectively prevented by administering ACR with APO and UMB. Sengul et al. (2021) have demonstrated that the ACR-infused AKI had considerably higher serum urea and creatinine levels. Therefore, APO, UMB, and their combination have preserved kidney functions due to their antioxidant effects.

Furthermore, the protective effects of APO and/or UMB in ACR-induced AKI have been confirmed by histological studies showing that in the ACR group, there was significant vacuolation and necrosis of the epithelial lining proximal and distal convoluted tubules, as well as marked splitting, shrinking, and hypercellularity of the glomerular tufts and capillary tufts. APO /or UMB improved the kidney against ACR-induced injury and significantly attenuated histopathological alterations in the kidneys, including reduced tubular necrosis, interstitial inflammation, and cast formation, as evidenced by the improved levels of the measured biomarkers and the histological changes. The combination treatment has shown enhanced protective effects compared to individual treatments alone, including a remarkably healthy appearance of the tissue, a decrease in the intensity of the blood vessel changes, reduced renal inflammation, and normalized histopathological damage.

Notably, the mechanism of ACR toxicity is not yet fully understood. However, studies have shown the involvement of oxidative stress cascades (Lujia Zhang 2023; Komoike and Matsuoka 2019). Multiple studies demonstrated that ACR and glycidamide can form covalent adducts with hemoglobin, DNA, and various protein functional groups like SH, causing chromosomal aberration and gene mutation that decreased endogenous antioxidant levels and increased ROS, which are crucial in ACR-induced nephrotoxicity (Koszucka et al. 2020; Wang et al. 2022b; Song et al. 2021). The antioxidants GSH and SOD are known to play a critical role in preventing oxidative damage (Sayed et al. 2022; Bin-Jumah et al. 2021). The current study found that renal oxidant/antioxidant status was severely disturbed by ACR by significantly decreasing GSH and SOD and increasing MDA as an indicator of lipid peroxidation. Alternatively, co-treatment of ACR with APO, UMB, and their combination significantly improved oxidant/antioxidant balance, shown by increasing GSH content and SOD activities along with the reduction of MDA. These findings are consistent with earlier research demonstrating the strong antioxidant benefits of APO, UMB, and their combination against different animal models (Asgharpour et al. 2020; Germoush et al. 2018).

To investigate the underlying molecular processes of the antioxidant effects of APO, UMB, and their combination against ACR-induced oxidative injury, Nrf2/HO-1 signaling was investigated. Interestingly, the transcription factors Nrf2 activation and HO-1 induction in AKI are part of an adaptive response aimed at restoring redox balance, reducing oxidative damage, suppressing inflammation, and promoting cell survival (Zhai et al. 2023; Hong et al. 2010). There have been reports that administrating ACR can disrupt the Nrf2 pathway, which in turn causes the expression of HO-1 to be downregulated (Zhao et al. 2017). This may weaken the antioxidant defenses within cells and make it more difficult for them to defend against inflammatory assaults and oxidative stress. Our findings showed that the administration of ACR led to a significant decrease in renal Nrf2/HO-1 expression. Nevertheless, simultaneous 10-day treatment with APO, UMB, and their combination greatly increased the expressions of Nrf2 and HO-1. Collectively, our findings demonstrated that APO, UMB, and their combination prevented renal oxidative damage brought on by ACR via modifying the Nrf2/HO-1 signaling pathway.

Then, the impact of inflammation on ACR-induced renal injury and the underlying molecular mechanism were investigated. TNF-α is a pro-inflammatory cytokine involved in immune responses and inflammation. It plays a crucial role in the pathogenesis of AKI by promoting inflammation and oxidative stress (Fatani et al. 2018). Inhibition of TNF-α can attenuate inflammatory responses and protect against renal injury (Bhargava et al. 2013). In this investigation, the combination treatment has shown a more significant reduction in TNF-α expression, indicating more protection against ACR-induced AKI. This combination may act synergistically, targeting multiple inflammatory pathways and improving the overall protective effect. Mechanistically, the current study demonstrated, for the first time, that APO, UMB, and their combination prevented the activation of the NLRP3 inflammasome signaling pathway in ACR-induced AKI by targeting NLRP3, ASC, GSDMD, IL-1β, and caspase-1, which is being investigated as a potential therapeutic approach for various inflammatory conditions, including AKI. Various signals, including DAMPs such as oxidative stress and mitochondrial dysfunction, trigger the activation of NLRP3 inflammasome in AKI. Upon activation, the NLRP3 inflammasome assembles and recruits the adaptor protein ASC, leading to the activation of caspase-1. Then, caspase-1 cleaves pro-inflammatory cytokines IL-1β and IL-18 into their active forms, which promotes inflammation, rupture of the cell membrane, and pyroptosis, which contribute to renal injury (He et al. 2016; Alatshan and Benkő, 2021). GSDMD is a key executor of pyroptosis. GSDMD forms pores in the membrane to enable the release of IL-1β and to drive cell lysis through pyroptosis a pro-inflammatory form of cell death associated with NLRP3 inflammasome activation (Xia et al. 2021). Targeting GSDMD has shown potential for ameliorating the inflammation of diabetic kidney disease (Wang et al. 2022c). In the current study, we noted upregulation of the NLRP3 inflammasome and pro-inflammatory cytokines after administration of ACR. Importantly, on the other hand, APO, UMB, and their combination protected rats from ACR-induced AKI by downregulating the NLRP3 inflammasome and inhibiting the assembly and activation of the NLRP3 inflammasome complex, thereby reducing the release of IL-1β and subsequent inflammation via modulating key components of the NLRP3 inflammasome pathway, such as NLRP3 itself, ASC, GSDMD, IL-1β, and caspase-1. A growing amount of data indicates that APO and UMB inhibit NLRP3 inflammasome production in different animal models, which is in line with this conclusion. by diminishing the inflammation caused by the NLRP3 inflammasome's activation and preventing kidney cell death and severe damage because of their antioxidant and anti-inflammatory properties (Jin et al. 2019; Luo et al. 2018).

## Conclusions

This work showed that the pathophysiology of the ACR-induced AKI model may include the NLRP3 signaling pathway. Furthermore, the administration of APO or UMB markedly suppressed renal inflammation and oxidative stress, and reduced pyroptosis via regulation of Nrf-2/HO-1 and NLRP-3 inflammasome signaling pathways. Additionally, the combination of APO and UMB significantly augmented the antioxidant, anti-inflammatory, and protective activity against ACR-induced AKI, which is greater than that of individual drugs. It is advised that more clinical trials be conducted to investigate our findings.

## Supplementary Information

Below is the link to the electronic supplementary material.

Supplementary file1 (PDF 689 KB)

## Data Availability

This article contains all of the data generated or evaluated throughout the study.
