# Supplementary figures and images for: Renoprotective effects of apocynin and/or umbelliferone against acrylamide-induced acute kidney injury in rats: role of the NLRP3 inflammasome and Nrf-2/HO-1 signaling pathways
Source: Naunyn Schmiedebergs Arch Pharmacol. 2024 Jul 19;398(1):569–80. doi: 10.1007/s00210-024-03271-9 (PMC11787205; doi:10.1007/s00210-024-03271-9)

Caspase-1

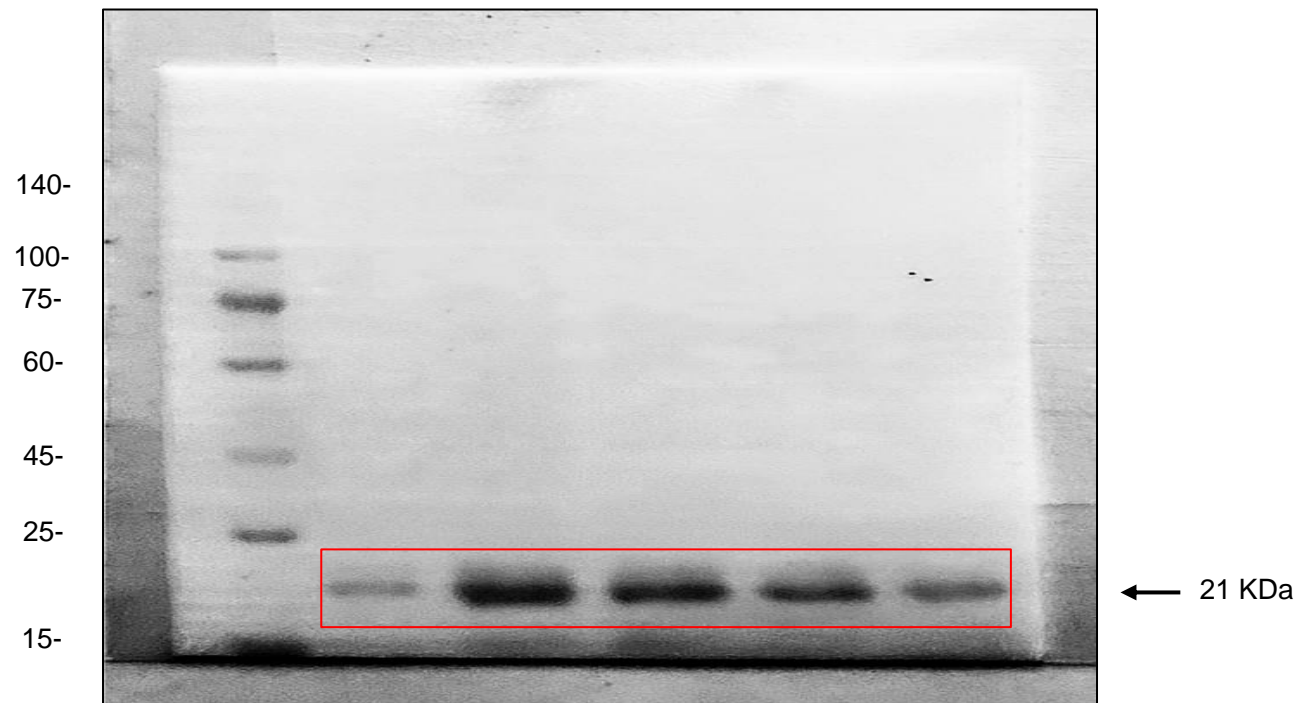

IL-1 $\beta$

100  
75-  
60-  
45-  
35-  
25-  
15-

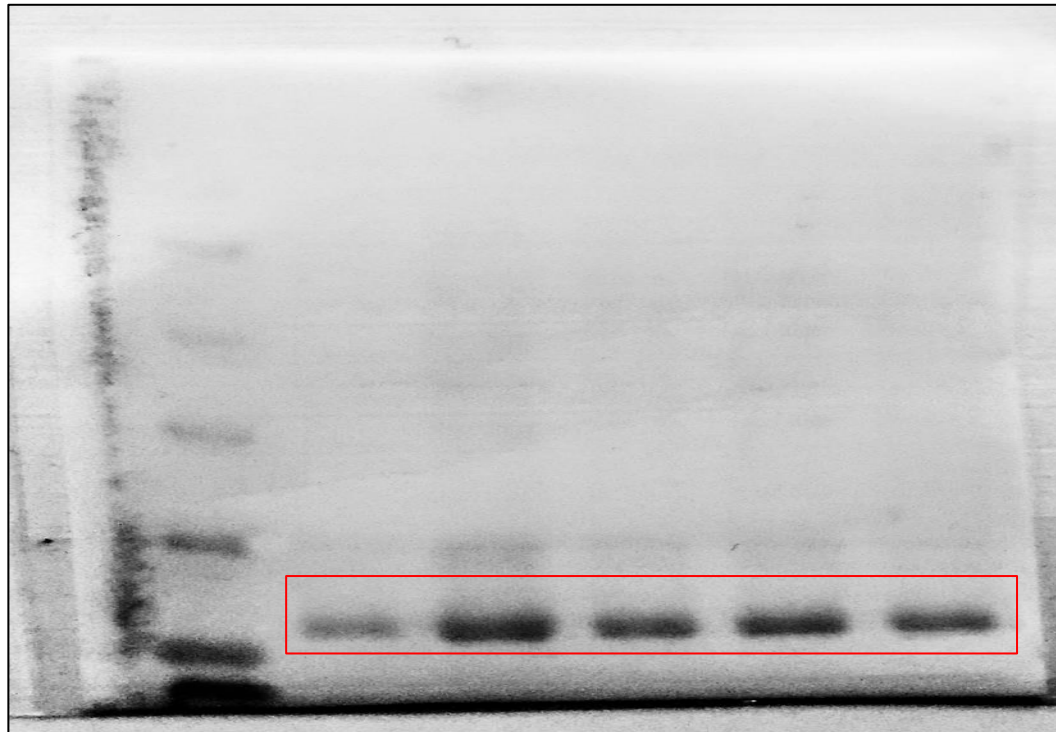

← 17 kDa

GSDMD

140-  
100-  
75-  
60-  
45-  
35-  
25-  
15-

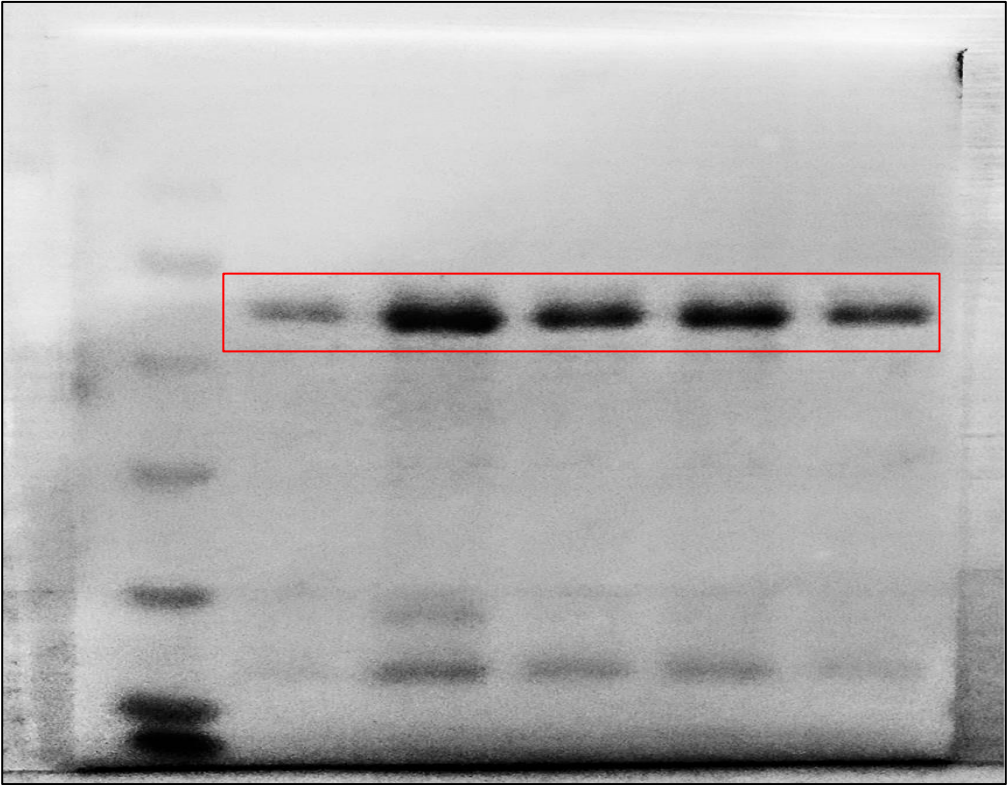

← 55 KDa

ASC

140-  
100-  
75-  
60-  
45-  
25-  
15-

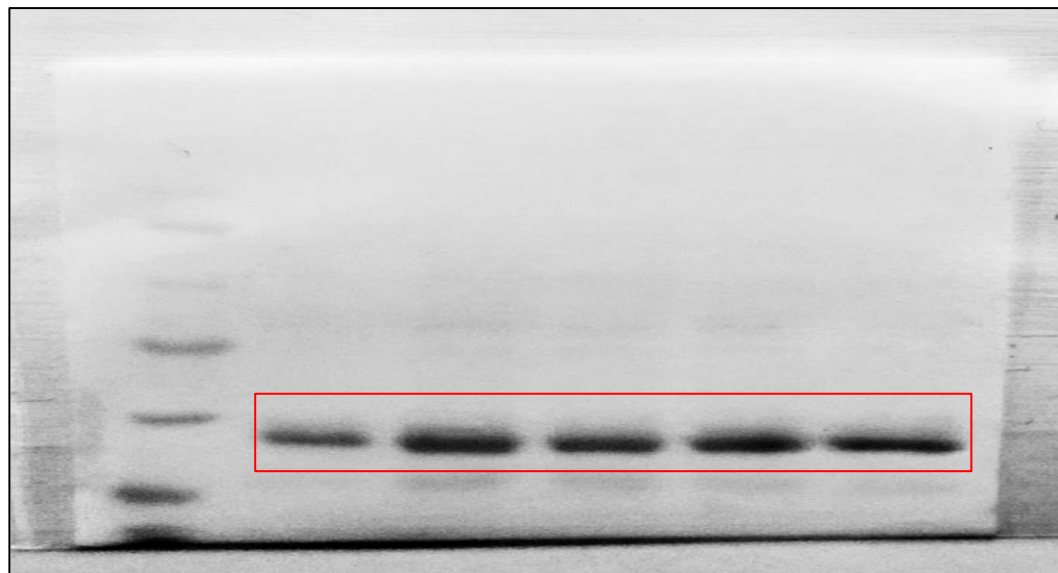

← 24 kDa

NLRP3

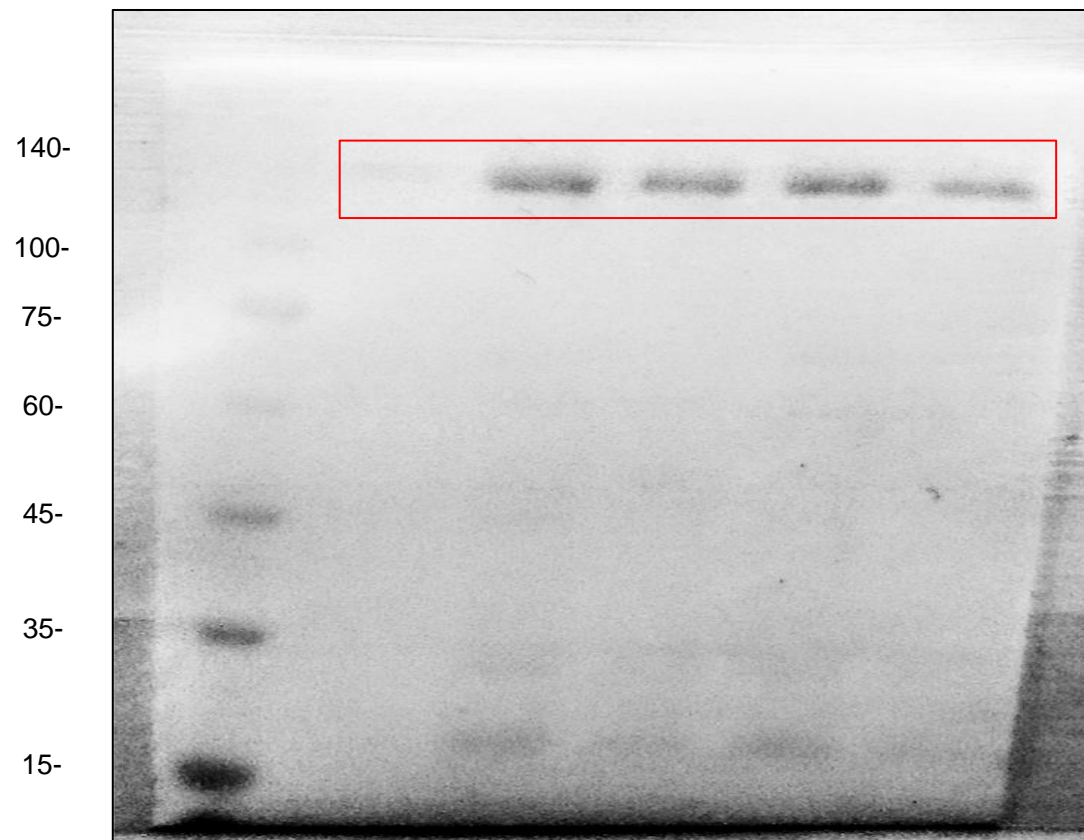

← 115 KDa

$\beta$ -actin

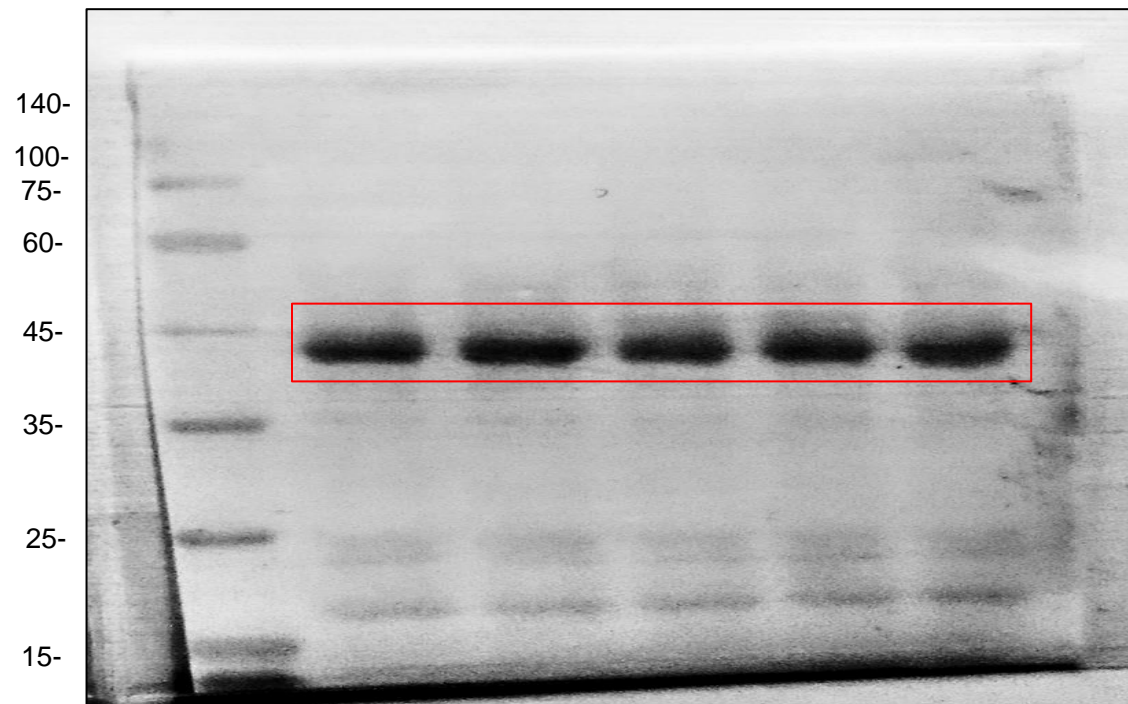

HO-1

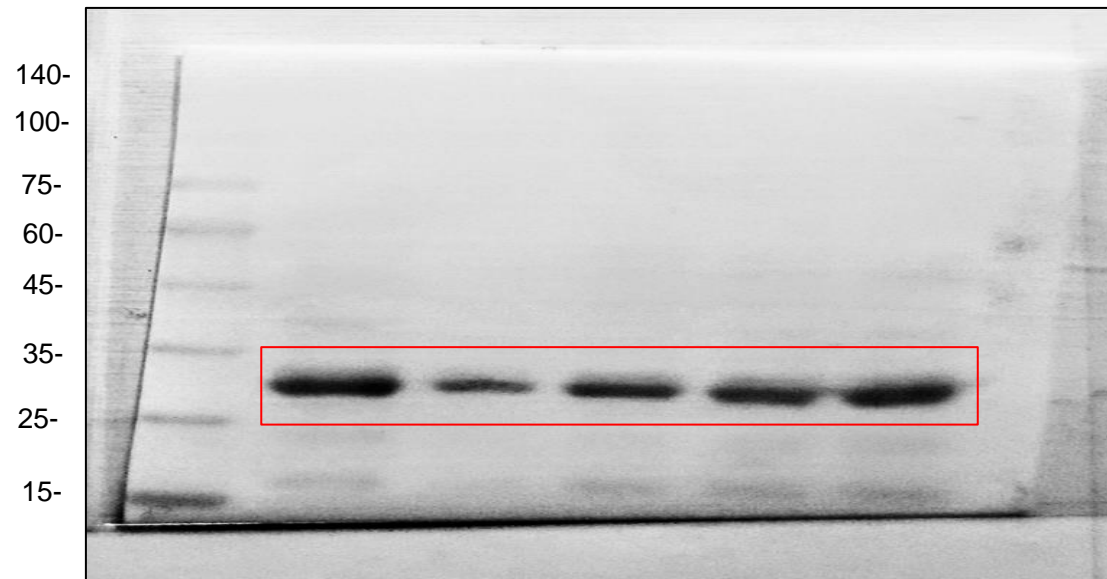

← 33 KDa

Nrf2

140-  
100-  
75-  
60-  
45-  
35-  
25-

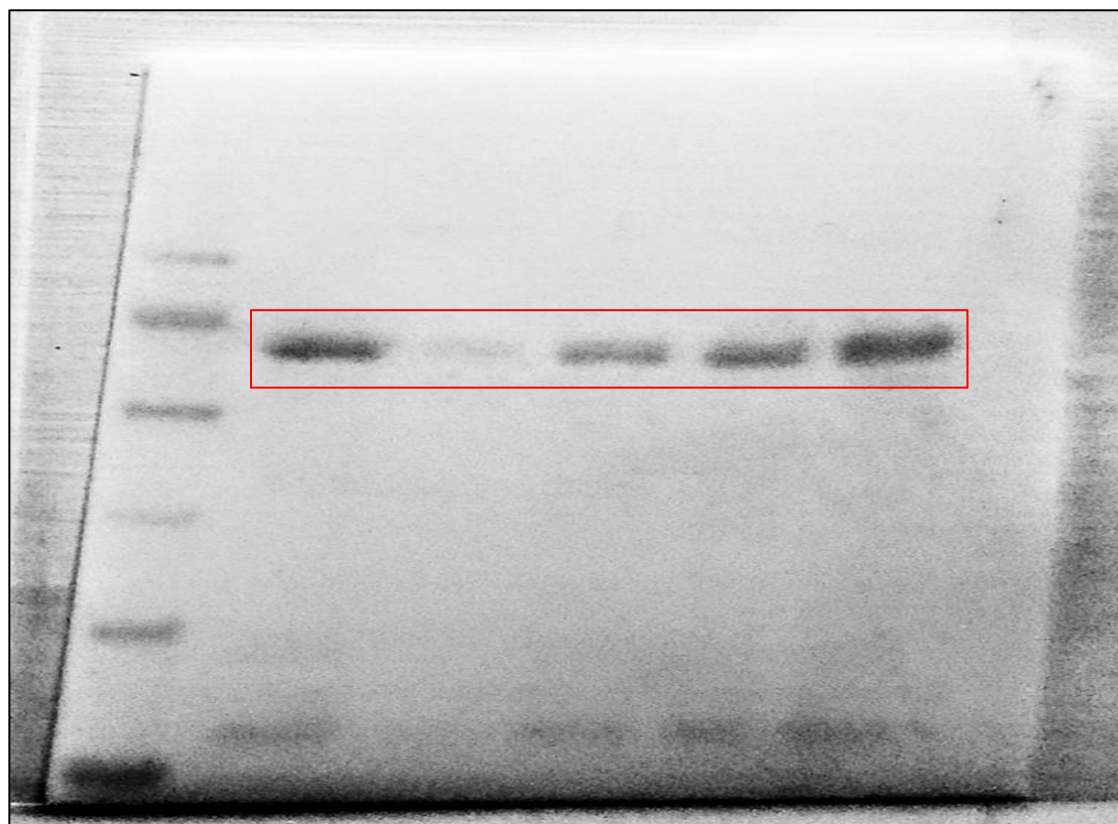

← 65 KDa

$\beta$ -actin

140-  
100-  
75-  
60-  
45-  
35-  
25-  
15-

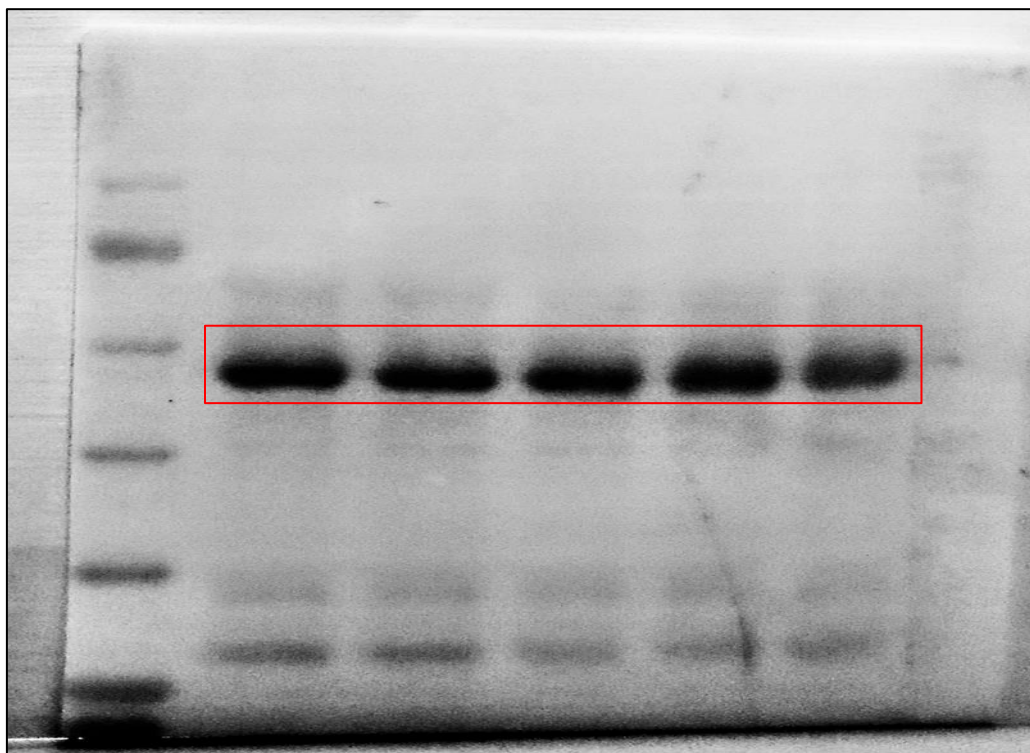

← 43 KDa

Supplement: Supplementary file 1 — Supplementary file1 (PDF 689 KB) [file 210_2024_3271_MOESM1_ESM.pdf]
